# Retraction: Role of alternative splicing of VEGF-A in the development of atherosclerosis

**DOI:** 10.18632/aging.204732

**Published:** 2023-05-15

**Authors:** Naishi Zhao, Jianfeng Zhang

**Affiliations:** 1 Department of Cardiovascular Surgery, Shanghai Chest Hospital, Shanghai Jiao Tong University, Shanghai, 200030, China

**Keywords:** atherosclerosis (AS), macrophage, endothelial cells, SRPK1, alternative splicing of VEGF, ApoE (-/-), high fat diet (HFD)

**This article has been retracted:** Aging has completed its investigation of this paper. We found internal duplication and, although cropped differently, three tube formation assay images in Figure 5G were previously published as Figure 3C in a much older paper by different authors [1]. As a result, both authors agreed that the article should be retracted.

The Administration of the Shanghai Chest Hospital, Shanghai Jiao Tong University was notified about the retraction by Aging Journal.

Original article: Aging. 2018; 10:2695–2708. . 

## References

[1] Naik MU, Mousa SA, Parkos CA, Naik UP. Signaling through JAM-1 and alphavbeta3 is required for the angiogenic action of bFGF: dissociation of the JAM-1 and alphavbeta3 complex. Blood. 2003; 102:2108–14. 10.1182/blood-2003-04-1114 12750158

